# Research on Multi-Scale Spatio-Temporal Graph Convolutional Human Behavior Recognition Method Incorporating Multi-Granularity Features

**DOI:** 10.3390/s24237595

**Published:** 2024-11-28

**Authors:** Yulin Wang, Tao Song, Yichen Yang, Zheng Hong

**Affiliations:** 1 College of Intelligent Transportation, Chongqing Vocational College of Public Transportation, Chongqing 402260, China; 2008wxwyl@163.com (Y.W.); 18716482190@163.com (Z.H.); 2 College of Electrical and Electronic Engineering, Chongqing University of Technology, Chongqing 400054, China; yyc123@stu.cqut.edu.cn

**Keywords:** graph convolutional network, behavior recognition, multi-granularity, bias weighting

## Abstract

Aiming at the problem that the existing human skeleton behavior recognition methods are insensitive to human local movements and show inaccurate recognition in distinguishing similar behaviors, a multi-scale spatio-temporal graph convolution method incorporating multi-granularity features is proposed for human behavior recognition. Firstly, a skeleton fine-grained partitioning strategy is proposed, which initializes the skeleton data into data streams of different granularities. An adaptive cross-scale feature fusion layer is designed using a normalized Gaussian function to perform feature fusion among different granularities, guiding the model to focus on discriminative feature representations among similar behaviors through fine-grained features. Secondly, a sparse multi-scale adjacency matrix is introduced to solve the bias weighting problem that amplifies the multi-scale spatial domain modeling process under multi-granularity conditions. Finally, an end-to-end graph convolutional neural network is constructed to improve the feature expression ability of spatio-temporal receptive field information and enhance the robustness of recognition between similar behaviors. The feasibility of the proposed algorithm was verified on the public behavior recognition dataset MSR Action 3D, with a accuracy of 95.67%, which is superior to existing behavior recognition methods.

## 1. Introduction

As an extremely important component in the field of computer vision, research on behavior recognition has always attracted considerable attention and has been widely applied. It has broad application prospects in intelligent surveillance, smart transportation, human–computer interaction, and other areas [1,2,3]. At present, human skeleton behavior recognition based on deep learning is mainly divided into three categories. The first type is to use convolutional neural networks [4,5,6] to model skeleton data as pseudo images, extracting highly abstract skeletal structural features. The second type is to use recurrent neural networks [7,8,9,10] to model skeleton data as sequences of coordinate vectors, capturing the dynamic correlations between consecutive frames of skeletal data to predict behavior categories. The last type is the graph convolutional network (GCN), which represents the human skeleton sequence as a spatio-temporal topological graph. By utilizing graph convolution, it effectively extracts the global features of the skeleton’s spatial structure, thereby enabling better modeling of the spatio-temporal characteristics of human skeleton information. Therefore, graph convolution-based human skeleton behavior recognition methods have become a research hotspot in recent years.

Yan et al. [11] introduced spatial–temporal graph convolutional networks (ST-GCNs), which for the first time utilized graph convolutional networks to model human skeleton data and achieved good recognition results in the process of action recognition. Shi et al. [12] proposed adaptive graph convolution, which calculates the similarity between joints based on input skeleton data of different action classes to adaptively measure the degree of correlation between joints. Li et al. [13] proposed a motion structure that emphasizes the dependency relationship between non-adjacent joints in space through action-linking modules and structural linking modules. References [14,15,16] proposed a multi-scale spatial graph convolutional network to capture feature information between joints in a wider space, using high-order polynomials of the adjacency matrix to aggregate features between remote joints. However, these methods have bias weighting issues in the process of spatial domain modeling, which means that in the process of modeling spatial position relationships using high-order adjacency matrices, joints far from the target joint make little contribution to recognition, and the final recognition result will be dominated by joints from local body parts. Meanwhile, due to the presence of information from different modalities and spatio-temporal scales within the skeleton, all of which are crucial for behavior recognition, many works have attempted to explore and utilize this information. Shi et al. [12] added the inter-frame difference between the bone flow and keypoint flow in 2s-AGCN as information for keypoint motion flow and bone motion flow. The Shift-GCN network proposed by Cheng et al. [17] performs more processing on the original data, extracting frame differences as dynamic information between keypoints and bones based on keypoint coordinates and bone vectors, and these four different forms of data are used as inputs to jointly predict category features.

Li et al. [18] performed higher-order transformations on the original skeleton data and employed a multi-stream network to fuse high-order information such as joint and bone information at the decision level, further enhancing the model’s performance. However, these methods did not take into account spatial granularity characteristics during human behavior. From the perspective of human kinematics, the recognition of certain actions relies on the characteristics between distant joints, while the identification of other similar actions is more dependent on subtle movement differences between local joints.

To address the aforementioned issues, a multi-scale spatio-temporal graph convolutional method integrating multi-granularity characteristics was proposed for human action recognition. The input skeleton data were initialized into data streams of different granularities to guide the network in learning the differences between similar actions. Additionally, a cross-scale fusion module was constructed for feature fusion among different granularities. By adopting a method of constructing multi-scale adjacency matrices, the adjacency matrices at different spatial scales were subtracted from each other to build sparse adjacency matrices, thereby solving the problem of biased weighting in the process of multi-scale spatial modeling. An end-to-end multi-scale graph convolutional network integrating multi-granularity characteristics was then constructed, and the feasibility of the proposed algorithm was validated on the MSR Action 3D dataset, which is publicly available for action recognition.

## 2. Skeleton-Based Behavior Recognition Based on Graph Convolution Networks

This section begins by introducing the fundamental principles of ST-GCNs for skeleton-based behavior recognition. Subsequently, it analyzes the bias weighting issue in graph convolutional networks, where the extraction of spatial features from skeletons tends to prioritize adjacent joints, making it difficult to capture dependencies between distant joints. Additionally, a solution to this problem is then proposed.

### 2.1. Spatio-Temporal Graph Convolutional Network

A GCN is widely used in the modeling of human skeleton data. In this method, the human skeleton is generally represented as a spatio-temporal graph G=(V,E) with N joints and T frames, where V represents the joints of the skeleton and E represents the edges connecting the human joints. The skeleton coordinates of human actions can be expressed as $X\in R^{C\times T\times N}$, where C represents the number of channels, T represents the number of frames in the video, and N is the number of joints in the human skeleton. The GCN-based model mainly consists of two parts: spatial graph convolution and temporal convolution.

In the spatial dimension, the feature extraction of any joint $v_{ti}$ in the skeleton graph by graph convolution operation is expressed as

(1) $$f_{out}(v_{ti})=\sum_{v_{tj}\in B(v_{ti})}\frac{1}{Z_{ti}(v_{tj})}f_{in}(p(v_{tj},v_{ti}))\cdot w(v_{tj},v_{ti})$$

where $f_{in}$ and $f_{out}$ represent the input and output features, respectively; $B(v_{ti})=\left\{v_{tj}\left|r(v_{tj},v_{ti})\right.\in R\right\}$ represents the set of neighboring joints of $v_{ti}$; and R controls the range of neighboring joints selected. $Z_{ti}(v_{tj})=\left|\left\{v_{tk}\left|l_{ti}(v_{tk})=l_{ti}(v_{tj})\right.\right\}\right|$ is the normalization term, and w is the weighting function of neighboring joints.

The operation of graph convolution in the time domain can be extended from graph convolution in the spatial domain, by using parameter Γ as the size of the temporal convolution kernel and it serving as a control for the temporal range of the neighbor set. Due to the introduction of temporal dimension information, the neighbor set in both spatial and temporal dimensions can be expressed as

(2) $$B(v_{ti})=\left\{v_{aj}\left|d(v_{tj},v_{ti})\right.\leq K,\left|a-t\right|\leq \Gamma /2\right\}$$

The corresponding label-mapping set for its neighboring joints is

(3) $$l_{ST}(v_{qj})=l_{ti}(v_{tj})+(a-t+\Gamma /2)\times K$$

where $l_{ti}(v_{tj})$ represents the label mapping for $v_{ti}$ in the case of a single frame.

Therefore, on the skeleton input defined by feature X and graph structure A, the output of the network after passing through a layer of graph convolution can be represented as

(4) $$f_{out}=\sigma (D^{-\frac{1}{2}}\tilde{A}D^{-\frac{1}{2}}f_{in}W)$$

In the formula, $\tilde{A}=A+I$ represents the skeleton graph structure of the human body, and the connection relationship between joints in the skeleton graph is represented by an N×N adjacency matrix A and an identity matrix I; D is the joint degree matrix, which is a diagonal matrix with diagonal elements indicating the number of edges connected to each joint, $D^{-\frac{1}{2}}\tilde{A}D^{-\frac{1}{2}}$ represents the normalized skeleton structure, W represents the learnable weight matrix of the network, and σ(⋅) denotes the activation function.

### 2.2. Analysis of Bias Weighting Problem Methods

The existing methods use high-order polynomials of adjacency matrices to aggregate multi-scale spatial structural information at different moments. Based on Formula (4), the iteration rule for high-order matrices is as follows:

(5) $$f_{out}=\sigma \left(\sum_{k=0}^{K}D_{\left(k\right)}^{-\frac{1}{2}}{\tilde{A}}^{k}D_{\left(k\right)}^{-\frac{1}{2}}f_{in}W_{\left(k\right)}\right)$$

where K is the highest power of the adjacency matrix and ${\tilde{A}}^{k}$ represents the *k*-th power matrix of $\tilde{A}$.

The K-order adjacency matrix in a graph convolutional network represents the existence of K paths between two joints.

As can be seen from the above equation, due to the existence of cycles between joints, there are more paths to joints that are closer to the current joint (with a distance less than K) than to joints that are exactly K steps away. It results in the network assigning greater weight to joints that are closer in distance during the network iteration. Therefore, when conducting multiscale modeling in the spatial domain, the aggregated features will be dominated by the motion information of local body parts, making it difficult for the network to effectively capture the dependencies between distant joints. We refer to this phenomenon as the bias weighting issue. Since some human behaviors involve coordinated movements between distant joints, it is evident that the bias weighting phenomenon is detrimental to the recognition of such behaviors.

To address the bias weighting issue mentioned above, reference [19] proposes a multi-scale adjacency matrix; the construction method of the adjacency matrix is redefined as follows:

(6) $${\left[{\tilde{A}}_{\left(k\right)}\right]}_{i,j}=\left\{\begin{array}{l} \;1\;\;\mathrm{if}\;\;d(v_{i},v_{j})=k,\\ \;1\;\;\mathrm{if}\;\;i=j,\\ \;0\;\;\mathrm{if}\;\;\mathrm{otherwise} \end{array}\right.$$

where $d(v_{i},v_{j})$ provides the shortest distance between two joints $v_{i}$ and $v_{j}$. By setting different values of *K*, we can obtain adjacency matrices of different scales. The K-order adjacency matrix formula can also be calculated using the following formula:

(7) $${\tilde{A}}_{\left(k\right)}=I+\vartheta ({\tilde{A}}^{k}\geq 1)-\vartheta ({\tilde{A}}^{k-1}\geq 1)$$

where $\vartheta ({\tilde{A}}^{k}\geq 1)$ represents assigning values greater than or equal to 1 in the matrix to 1; replacing ${\tilde{A}}^{k}$ in Equation (5) with ${\tilde{A}}_{\left(k\right)}$, we obtain

(8) $$f_{out}=\sigma \left(\sum_{k=0}^{K}D_{\left(k\right)}^{-\frac{1}{2}}{\tilde{A}}_{\left(k\right)}D_{\left(k\right)}^{-\frac{1}{2}}f_{in}W_{\left(k\right)}\right)$$

where $D_{\left(k\right)}^{-\frac{1}{2}}{\tilde{A}}^{k}D_{\left(k\right)}^{-\frac{1}{2}}$ represents the standardized K-order adjacency matrix. In this paper, we propose a method of subtracting the K-order adjacency matrix from the K-1 order matrix to eliminate the bias weighting problem that exists in the original modeling approach, which allows the model to better capture the relationships between features that are more dependent on distant joints for action categorization.

**Figure 1:**
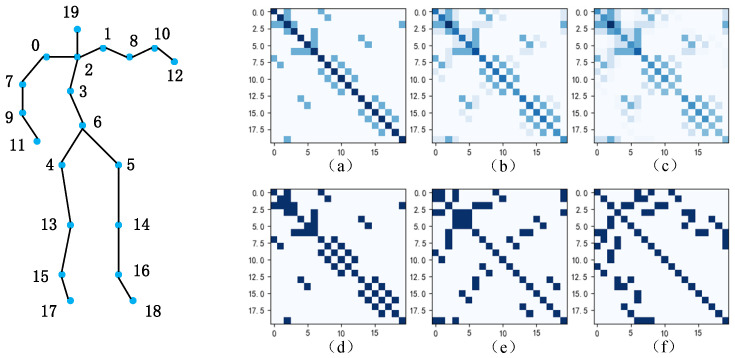
Adjacency matrix topology diagram. (**a**–**c**) respectively represent the topological graphs of first-order, second-order, and third-order adjacency matrices used to connect human skeletal joints, while (**d**–**f**) respectively represent the topological graphs after constructing multi-scale adjacency matrices.

As shown in Figure 1a–c represent the topological diagrams of the first-order, second-order, and third-order adjacency matrices used to connect human skeletal joints in a multi-scale spatial model. As the order of the adjacency matrix increases, joints closer to the current joint are assigned greater weights (the darker the color, the greater the weight assigned to the joint). Especially when introducing new joints to refine human skeletal features, the distance between the original two joints may increase due to the newly added joints; thus, the weights assigned to each other by the two joints will be further reduced. Figure 1d–f represent the topological graphs after constructing a multi-scale adjacency matrix; at this time, the adjacency matrix is reasonably sparsified, enabling the model to assign equal weights to distant joints and better capture the relationships between joints that are farther apart.

## 3. Improved Graph Convolutional Human Behavior Recognition Algorithm

This section begins by introducing the proposed behavior recognition approach based on a multi-scale spatio-temporal graph convolutional network (MS-TGCN) that incorporates multi-granularity features, along with an overview of the network’s overall architecture. Next, we present a multi-granularity skeleton segmentation strategy tailored for recognizing similar behaviors. Finally, a cross-scale feature fusion layer (CSFL) is designed to integrate multiple skeleton features of different granularities.

### 3.1. The Multi-Scale Spatio-Temporal Graph Convolution Network Incorporating Multi-Granularity Features

**Figure 2:**
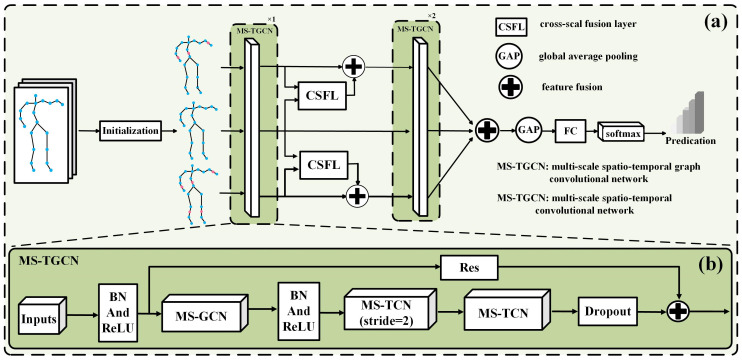
Framework of multi-scale spatio-temporal graph convolutional network model incorporating multi-granularity features. (**a**) represents the overall framework of the proposed network, and (**b**) represents the framework of the multi-scale spatio-temporal convolutional module.

In order to fully consider the granularity features of human behavior and leverage their advantages in different behavior recognition processes, this paper proposes a multi-scale spatio-temporal graph convolutional network incorporating multi-granularity features for human behavior recognition; the network model framework is shown in Figure 2. Firstly, joint information of the human body is initialized into data streams of different granularity sizes; considering some behaviors within the dataset are highly similar, it is necessary to refine the joint data to capture more subtle semantic information between behaviors. Secondly, the refined data is fed into the MS-TGCN block to extract its spatio-temporal features. Then, the obtained output features are fed into the CSFL to blend coarse and fine-grained features, to capture the differences in features between similar behaviors. Finally, the fused features are fed into the MS-TGCN layer to further extract their spatio-temporal features and obtain the classification results.

The framework of the multi-scale spatio-temporal convolution network is shown in Figure 2b. Firstly, the normalized multi-granularity data stream is fed into the MS-TGCN, and the topological relationships between joints are reconstructed in the spatial domain using the multi-scale adjacency matrix mentioned above. By setting different K values, spatial feature fusion is performed on joints at different distances. Secondly, the data are input into two multi-scale time convolutional layers with different step sizes to capture broader temporal contextual features. Finally, the residual module is used to connect the input and output. The MS-TCN and MS-GCN modules utilized in this context correspond to the respective modules mentioned in reference [19].

### 3.2. Fine-Grained Skeleton Construction Strategy

**Figure 3:**
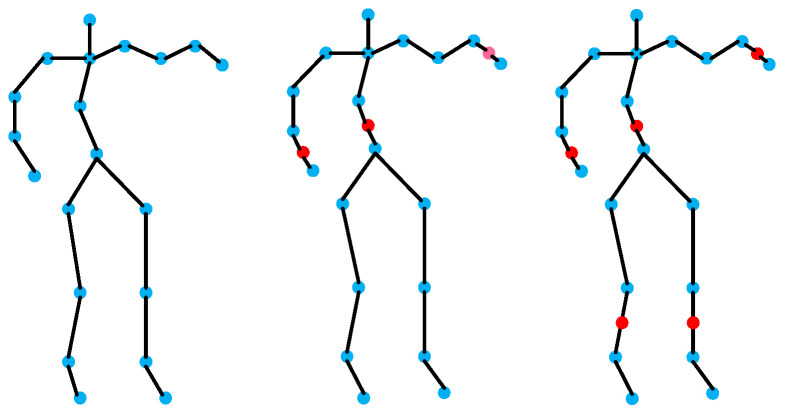
Three granularity representation methods for MSR Action 3D. The blue nodes represent the original coarse-grained joints, and the red nodes represent the newly added fine-grained joints.

Due to the high degree of overlap between similar behaviors in the spatio-temporal domain, traditional graph convolutional models have difficulty in capturing the semantic information that truly distinguishes categories and in learning accurate representations. In order to accurately depict fine-grained human behavior, this paper introduces a multi-granularity feature-learning method, initializing the human skeleton map into different fine-grained levels (a fine-grained skeleton refers to a human skeleton graph that is composed of more and finer joints.), as shown in Figure 3. This involves expanding the connections in coarse-grained graphs to tighter connections in fine-grained graphs, enabling fine-grained graphs to represent refined semantic information.

A single joint in the fine-grained skeleton graph is supplemented by averaging multiple adjacent joints in the coarse-grained skeleton graph using a two-dimensional average pooling method. The overall representation of the fine-grained skeleton graph is then obtained through concatenation operations. The formula for multi-granularity initialization is expressed as

(9) $$V_{ck}=pooling(V_{f_{1}}+V_{f_{2}}+\cdots +V_{f_{h}}),k\leq h$$

(10) $$Graph_{new}=concat(V_{c_{1}},V_{c_{2}},\cdots ,V_{c_{k}})$$

where $V_{ck}$ represents the joint information of k supplemented joints in the fine-grained skeleton graph, $V_{f_{h}}$ represents the joint information of h joints in the fine-grained skeleton graph, and $Graph_{new}$ represents the physical skeleton of the fine-grained graph.

### 3.3. Cross-Scale Feature Fusion

**Figure 4:**
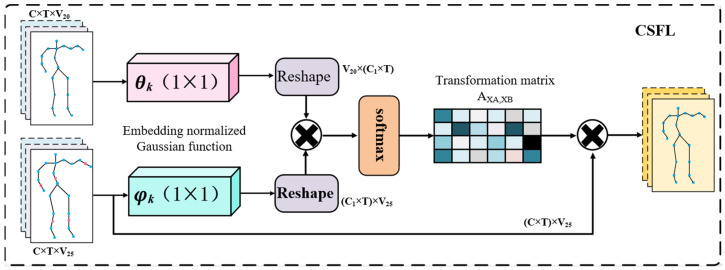
The structure of cross-scale feature fusion layer (CSFL).

To achieve feature fusion between a coarse and fine granularity, fine-grained features are used to guide the original granularity features to learn discriminative feature expressions between similar behaviors; inspired by reference [12], this paper proposes an adaptive cross-scale feature fusion module, as shown in Figure 4.

Namely, this means embedding a normalized Gaussian function in the network to calculate the feature-mapping relationship between two sizes and generate a cross-scale feature fusion matrix. The specific operation is as follows:

(11) $$f(v_{i},v_{j})=\exp\left[\psi^{T}(v_{i})\theta (v_{j})\right]/\sum_{j=1}^{N}\exp\left[\psi^{T}(v_{i})\theta (v_{j})\right]$$

where $\psi^{T}(v_{i})=W_{\psi }v_{i}$ and $\theta (v_{j})=W_{\theta }v_{j}$ represent embedded operations, while $W_{\psi }$ and $W_{\theta }$ are the corresponding weight parameters.

Taking 20-joint coarse-grained skeleton information and 25-joint fine-grained skeleton information as examples, the feature dimensions of the two input granularity features $f_{1}$ and $f_{2}$ are $C\times T\times V_{20}$ and $C\times T\times V_{25}$, respectively, where C represents the number of channels of the embedded Gaussian function. A 1 × 1 convolution operation is performed on both of the two granularity features, then dimension transformation is applied to both results, followed by matrix multiplication. Finally, an adaptive transformation matrix is obtained through a softmax classifier as follows:

(12) $$A_{f_{1},f_{2}}=softmax(f_{1}^{T}W_{\psi }^{T}W_{\theta }f_{2})\in [0,1]$$

This adaptive transformation matrix can dynamically adjust the mapping relationship between different granularity features, and the fused feature $\tilde{X_{out}}$ after scale fusion can be represented as

(13) $$\tilde{X_{out}}=\lambda GCN(A_{f_{1},f_{2}},f_{2})+f_{1}$$

$GCN(A_{f_{1},f_{2}},f_{2})$ represents the fused features obtained through the graph convolution operation using the transformation matrix $A_{f_{1},f_{2}}$ on a 25-joint scale. Studies have shown that the output feature maps from the shallow layers of the network can improve the quality of semantic segmentation and capture finer details [20,21]. This is because the deep feature maps of the graph convolution network often focus on high-level semantic information, while the local detail information of various skeleton parts usually exists in the shallow features; as the network goes deeper, these local details are gradually destroyed or even completely lost. Therefore, we choose to perform cross-scale feature fusion after a multi-scale graph convolution of the data, and introduce a hyperparameter λ in the fusion process to adjust the fusion ratio reasonably.

## 4. Experiment and Result Analysis

### 4.1. Experimental Dataset

The MSR Action 3D dataset contains the 3D coordinates of 20 human skeleton joints collected by a c Kinect v1 depth camera from Microsoft, USA. It consists of 10 subjects each performing 20 actions, with each action repeated 2 to 3 times. The number of frames in each action sequence ranges from 10 to 100, resulting in a total of 567 action sequence sample data. Due to the presence of highly similar actions in this dataset, it serves as an excellent benchmark to validate the effectiveness of the algorithm proposed in this paper. A cross-validation method based on subject classification is used to test the performance of the model, where subjects 1, 3, 5, 7, and 9 are used for training and subjects 2, 4, 6, 8, and 10 are used for testing.

### 4.2. Experimental Environment and Settings

This experiment is implemented based on a multi-scale spatio-temporal graph convolutional network that incorporates multi-granularity features, as shown in Figure 2. The benchmark network is a stacked three-layer multi-scale spatio-temporal graph convolutional network, with input and output channels of (3, 96), (96, 192), and (192, 384), and “initialization”, representing fine-grained data initialization; CSFL stands for the cross-scale fusion layer, GAP represents the global average pooling layer, and FC denotes the fully connected layer. The entire network is set with a batch size of 64 for the dataset, and it is trained for 150 epochs. The initial learning rate is set to 0.1, which is reduced to one-tenth at epochs 80 and 120. The dropout rate is set to 0.25, and the weight decay is set to 0.0001. These training parameters are set with reference to comparative algorithms to facilitate comparisons in the accuracy of action recognition, and they also align with the standard setting conventions for neural network training.

### 4.3. Experimental Results and Analysis

#### 4.3.1. Comparative Experiment Using Unbiased Weighting Method

To verify the effectiveness of the proposed multi-scale adjacency matrix method, this paper designs an experiment to compare the performance differences of the model before and after the introduction of this method. The experiment uses a stacked three-layer MS-TGCN network, where MS-TGCN-D represents multi-scale spatio-temporal graph convolution after applying the multi-scale adjacency matrix method, and the maximum value of the adjacency matrix for spatial positional relationships in the MSR Action 3D dataset is set to K = 10.

As shown in Table 1, when only using the MS-TGCN network, the accuracy of behavior recognition roughly shows a decreasing trend with a continuous increase in K value, which well proves the bias weighting problem caused by using high-order adjacency matrices. When using the MS-TGCN-D network, the introduction of the multi-scale adjacency matrix method brings a 2.76% improvement to the network at K = 6. For other values of K, it can also bring improvements ranging from 0.19% to 0.79%, thus verifying the effectiveness of introducing a multi-scale adjacency matrix. However, when K = 8 and K = 10, the accuracy of the network decreases by 0.72% and 0.39%, respectively; this is due to the highly similar characteristics of the action categories in the dataset, and the distant joints contribute little to the recognition performance of the network. If too large a K value is used, the network’s ability to capture the features of distant joints will increase, which does not align with the correlation between joints in most actions, thereby leading to a decrease in recognition accuracy. Therefore, in the subsequent experiments involving multi-granularity feature fusion, the value of K should not be too large; in this paper, K = 6 is selected for verification in the following experiments.

#### 4.3.2. Comparative Experiments on Fusing Multi-Granularity Features

**Figure 5:**
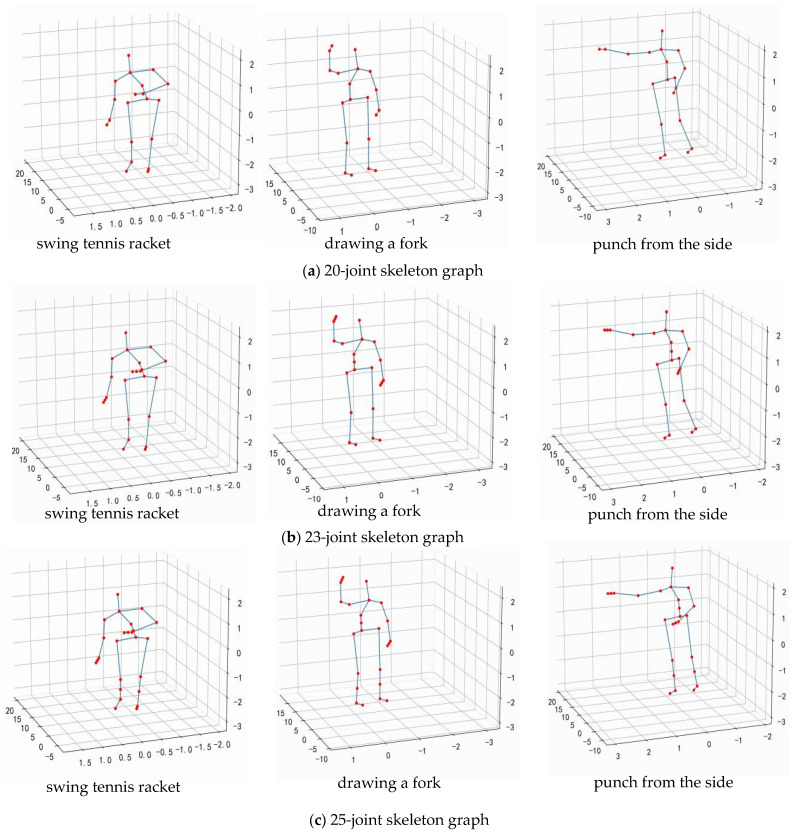
Skeleton graphs of different granularities.

The fine-grained features of human joint information can fully represent refined semantic features during human movement. The 20-joint skeleton data in the MSR Action 3D dataset are refined into 23 and 25 joints using the method proposed in Section 3.2, as shown in Figure 5. Then, comparative experiments are conducted on the skeleton data of different fine-grained levels.

The accuracy of behavior recognition using different granularities is shown in Table 2. The accuracy for each behavior is shown in Table 3. As can be seen from Table 2, using fine-grained skeletons with either 23 or 25 joints alone cannot improve the overall behavior recognition accuracy; instead, it decreases. This is because the value of K, the order of the adjacency matrix, was selected based on 20 joints. By comparing Table 3, it can be seen that fine-grained data can effectively distinguish some similar behaviors (such as drawing a fork, drawing a circle, and drawing a tick); the accuracy of punching from the side has increased from 86.7% to 100%; and the accuracy improvement in bending action is the highest, reaching 19.5%. This is because the inserted joints are the waist, wrist, and calves; inserting the wrist joints allows the model to capture the differences in motion feature between drawing a fork, drawing a circle, and drawing a tick, while inserting the waist joints helps the model capture the feature expression during the bending process. However, the recognition accuracy of this model has decreased for some other behaviors (such as high waving, overhand serve, and pounding). This is because these actions rely heavily on the movement state of the entire arm, and the inserted joints make it easier for the network to capture the movement differences at the front end of the arm, resulting in a decrease in the ability to capture the motion state of the arm near the torso.

The CSFL proposed in this paper blends different granularity features, allowing the network to fully integrate fine-grained features on the basis of its original performance, thereby improving network performance. The cross-scale feature fusion experiment adopts three settings: fusing 20 joints with 23 joints and 25 joints, respectively, as well as fusing all three simultaneously. In these experiments, the CSFL is integrated into the backbone network (MS-TGCN-D) where different fusion ratio parameters can balance the influence between coarse-grained and fine-grained. To verify the impact of fusing different granularity data under different fusion ratio parameters on the network performance, this paper conducted comparative experiments on the values of λ and the fusion methods. The experimental results are shown in Table 4. According to the experimental results, it can be seen that when the network fuses three types of granularity data and the fusion ratio parameter is set to 0.1, the recognition accuracy reaches 95.67%, which is 0.79% higher than the accuracy without multi-granularity fusion. At this point, the network performance is optimal. This fully demonstrates the effectiveness of a neural network that fuses multi-granularity features.

**Figure 6:**
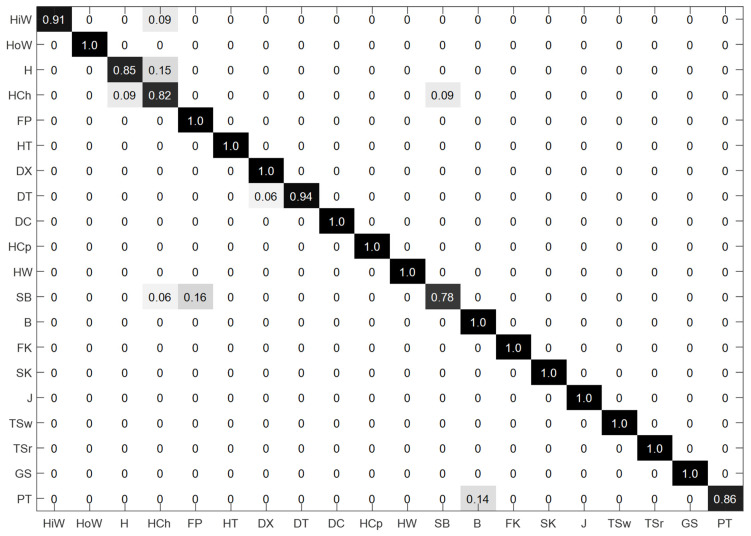
The confusion matrix of the MSR Action 3D dataset. The darker the background color of each grid in the figure, the higher the recognition rate it represents.

With a network recognition accuracy of 95.67%, the confusion matrix of the MSR Action 3D dataset is shown in Figure 6. As shown in the figure, the multi-scale spatio-temporal graph convolutional network that incorporates multi-granularity features can improve the accuracy of some similar behaviors, such as drawing a fork, drawing a circle, picking up and throwing, bending down, and swinging tennis rackets on the basis of the original network; in particular, the recognition accuracy of bending actions has reached 100%, which is a significant improvement compared to the original network. However, the accuracy for some behaviors, such as hammering, hand catching, and raising your hand high, have decreased. This is related to the positions of the joints inserted at different levels of granularity. Different actions require fine expressions from joints in different parts of the body. The proposed multi-granularity approach in this paper mainly involves adding additional joints at the wrists, waist, and lower legs, which is why the discrimination accuracy for actions such as drawing crosses, drawing circles, picking up and throwing, bending over, and swinging a tennis racket have improved. However, for actions like punching, grabbing, and high waving that primarily involve finger and arm movements, our method did not insert more joints in these areas, making it difficult to finely express and distinguish between these types of actions. Instead, the insertion of joints in other parts of the body led to a dilution of the feature weights for the fingers and arms, ultimately resulting in a decrease in the recognition accuracy for these types of actions.

#### 4.3.3. Comparison Experiment with Other Models

In order to better verify the improvement of the model in behavior recognition performance, this paper compared and analyzed its recognition accuracy with existing behavior recognition methods on the MSR Action 3D dataset. The comparison results are shown in Table 5.

The multi-scale spatio-temporal graph convolutional network proposed in this paper, which integrates multi-granularity features, achieves a behavior recognition accuracy of 95.67% on the MSR Action 3D dataset, and its experimental results are superior to most existing behavior recognition methods. Compared with the methods proposed in references [20,22], the accuracy has been improved by 2.04% and 3.77%, respectively; compared with the adaptive skeleton center point method proposed in reference [21], the accuracy has been improved by 7.2%; compared with the method of combining graph convolution with Long Short-Term Memory (LSTM) networks [10] and the multi-view depth motion map method STACOG [23], the accuracy is improved by 1.17% and 2.27%, respectively; compared with the enhanced data-driven algorithm proposed in reference [24] and the method of using point cloud data as input for behavior recognition [25], the accuracy has been improved by 0.86% and 0.49%, respectively; and compared with the fusion multi-modal data feature method proposed in reference [26], the accuracy has been improved by 3.76%. By comparison, it can be seen that the algorithm proposed in this paper has a high recognition accuracy in using 3D human skeleton information for human behavior recognition and also shows a strong competitiveness compared to existing methods.

## 5. Conclusions

Most human action recognition methods based on graph convolutional networks primarily extract global features from the human action process, and they often lack the ability to capture local differences between similar actions. The varying levels of granularity in human skeleton data can represent different hierarchical semantic characteristics during the action process, and fusing motion features at different granularity levels can effectively improve the network’s performance in recognizing similar actions.

This paper proposes a multi-scale spatio-temporal graph convolutional method that integrates multi-granularity features for human action recognition. By initializing human skeleton data into data streams of different granularities and employing a spatio-temporal graph convolutional network with multi-scale adjacency matrices, the network’s spatio-temporal representation capability is effectively enhanced. Additionally, an adaptive cross-scale fusion layer is introduced to guide the model in learning discriminative feature representations between similar actions using fine-grained features, thereby improving the accuracy of the network in recognizing similar actions.

Experimental results on the MSR Action 3D dataset demonstrate that our proposed algorithm outperforms existing methods in terms of overall accuracy for different action recognition tasks, particularly showing significant improvements in the accuracy of recognizing some highly similar actions. However, there are still many challenges in the field of action recognition. For instance, when dealing with different populations, the skeletal spatial scales of the same action can vary significantly between adults and children, which can affect the model’s recognition accuracy. Normalizing the scale of skeletal spatial structure is an issue that needs to be considered in action recognition. Additionally, the difference in action speed also poses a challenge. Similar body movements performed at different speeds can correspond to different behaviors. For example, slow movements may represent Tai Chi exercises, while fast movements could indicate pushing and shoving or fighting. Considering the temporal fine-grained aspects of actions is another research direction for future action recognition.

## Figures and Tables

**Table 1:** Comparison of training accuracy using multi-scale adjacency matrix method (%).

Model Methods	The Value of K in K-Order Adjacency Matrix						
	K = 2	K = 3	K = 4	K = 5	K = 6	K = 8	K = 10
MS-TGCN	94.09	93.31	92.52	92.91	92.12	92.91	92.12
MS-TGCN-D	94.28	93.70	93.31	92.91	94.88	92.13	91.73

**Table 2:** Accuracy of recognition using different granularity data on MS-TGCN-D network.

Number of Joints/(Pieces)			Accuracy (%)
20	23	25	
✓			94.88
	✓		94.09
		✓	93.31

**Table 3:** Accuracy of each behavior’s recognition using data of different granularities.

MSR Action 3D Behavior Types	Accuracy of Behavior Recognition (%)		
	MS-TGCN-D (20 Joints)	MS-TGCN-D (23 Joints)	MS-TGCN-D (25 Joints)
Raise your hand high (HiW)	100	81.8	81.8
Wave your hand in front of your chest (HoW)	100	100	100
Hammering (H)	92.3	75.0	75.0
Hand catch (HCh)	100	100	100
Forward punch (FP)	100	94.2	90.9
High throw (HT)	100	97.6	88.9
Drawing a fork (DX)	92.3	100	100
Drawing a tick (DT)	100	100	100
Drawing a circle (DC)	93.8	93.8	93.8
Hand clap (HCp)	100	100	100
Two-hand wave (HW)	100	100	100
Punch from the side (SB)	86.7	100	100
Bending down (B)	58.3	77.8	77.8
Kick korward (FK)	100	100	100
Kick sideways (SK)	100	100	100
Jogging (J)	100	100	100
Swing a tennis racket (TSw)	93.8	92.1	83.3
Overhand serve (TSr)	100	95.9	93.8
Swing a golf club (GS)	100	100	100
Picking up and throwing (PT)	75	73.6	75

**Table 4:** Recognition accuracy of MS-TGCN-D (CSFL) model by fusing different numbers of joint points under different proportional parameters.

The Number of Joint Points			The Value of the Proportional Parameter λ	Accuracy (%)
20	23	25		
✓			\	94.88
✓	✓		0.1	94.09
			0.2	94.28
			0.3	93.70
✓		✓	0.1	93.31
			0.2	94.88
			0.3	92.92
✓	✓	✓	0.1	95.67
			0.2	92.92
			0.3	93.70

**Table 5:** Comparison of recognition accuracy with other methods on the MSR Action 3D dataset.

Method	Accuracy (%)
Yang et al. [20]	93.63
Ran et al. [21]	88.47
Agahian et al. [22]	91.90
Zhao et al. [10]	94.50
STACOG [23]	93.40
Zhang et al. [24]	94.81
Wu et al. [25]	95.18
You et al. [26]	91.91
Ours	95.67

## Data Availability

The data used to support the findings of this study are available from the corresponding author upon request.
